# Targeting MDMX and PKCδ to improve current uveal melanoma therapeutic strategies

**DOI:** 10.1038/s41389-018-0041-y

**Published:** 2018-03-29

**Authors:** R. C. Heijkants, M. Nieveen, K. C.’t Hart, A. F. A. S. Teunisse, A. G. Jochemsen

**Affiliations:** Department of Cell and Chemical Biology, Leiden University Medical Centre, Leiden, The Netherlands

## Abstract

Uveal melanoma (UM) is the most frequent ocular cancer in adults, accounting for ~5% of the total melanoma incidence. Although the primary tumor is well treatable, patients frequently develop metastases for which no curative therapy exists. Highly activated protein kinase C (PKC) is a common feature of UM and has shown potential as therapeutic intervention for UM patients. Unfortunately, PKC inhibition as single treatment appears to have only limited clinical benefit. Combining PKC inhibition with activation of p53, which is rarely mutated in UM, by MDM2 inhibitors has shown promising results in vitro and in vivo. However, clinical studies have shown strong adverse effects of MDM2 inhibition. Therefore, we investigated alternative approaches to achieve similar anticancer effects, but with potentially less adverse effects. We studied the potential of targeting MDMX, an essential p53 inhibitor during embryonal development but less universally expressed in adult tissues compared with MDM2. Therefore, targeting MDMX is predicted to have less adverse effects in patients. Depletion of MDMX, like the pharmacological activation of p53, inhibits the survival of UM cells, which is enhanced in combination with PKC inhibition. Also pan-PKC inhibitors elicit adverse effects in patients. As the PKC family consists of 10 different isoforms, it could be hypothesized that targeting a single PKC isoform would have less adverse effects compared with a pan-PKC inhibitor. Here we show that specifically depleting PKCδ inhibits UM cell growth, which can be further enhanced by p53 reactivation. In conclusion, our data show that the synergistic effects of p53 activation by MDM2 inhibition and broad spectrum PKC inhibition on survival of UM cells can also largely be achieved by the presumably less toxic combination of depletion of MDMX and targeting a specific PKC isoform, PKCδ.

## Introduction

Uveal melanoma (UM) is a collective name for a cancer arising from the melanocytes originating from the choroid (85%), iris (5%) or ciliary body (10%)^1^. Primary tumors can be treated effectively, but approximately half of the patients develop metastasis within 15 years after primary tumor detection^2,3^. Thus far, no therapeutic intervention has been successful in treating metastatic UM. Due to the lack of effective therapy, the median survival of patients with metastasized UM therefore ranges between 3 and 12 months.

UM is most frequently driven by activating mutations in the G-proteins GNAQ (50%) or GNA11 (43%)^4–6^. As a result, these G-proteins are locked in a guanosine-5'-triphosphate-bound state, continuously activating a number of signaling pathways, including the mitogen-activated protein kinase (MAPK) pathway. The latter is achieved via an important downstream effector of GNAQ and GNA11, phospholipase C-β, which hydrolyzes phosphatidylinositol 4,5-bisphosphate to generate inositol 1,4,5-trisphosphate and diacylglycerol^7^. These are both second messengers activating various protein kinase C (PKC) isoforms, which in turn fuel the continuous activation of the MAPK pathway. These findings have spurred studies to investigate the potential of PKC and MAPK/extracellular-signal regulated kinase (ERK) (MEK) inhibitors in treating UM patients. UM cells containing a GNAQ or GNA11 mutation are indeed dependent on MAPK signaling and were shown to be sensitive to both MEK and PKC inhibition^8,9^. However, pre-clinical in vivo studies showed that both MEK and PKC inhibition is needed to completely abolish MAPK signaling and thereby tumor growth^9^. Confirming these pre-clinical studies, phase I clinical trials show promising results, but only modest clinical benefit, for both PKC and MEK inhibitors as single agents^10^. Based on the pre-clinical studies, a phase II clinical trial was conducted to assess combined PKC and MEK inhibition (NCT01801358). This phase II clinical trial was terminated premature due to strong adverse effects^11^. Based on the clinical activity of PKC inhibitor Sotrastaurin/AEB071, progression-free survival of 15 weeks in half of the patients^10^ has encouraged us and others to explore whether the effect of Sotrastaurin can be boosted by interfering with additional oncogenic or tumor-suppressor pathways. New insights into UM has stimulated studies combing PKC inhibition with CDK inhibition or targeting the phosphatidylinositol-4,5-biphosphate 3 kinase/ mamalian target of rapamycin pathway^11^. An alternative interesting approach could be the activation of p53, which is essentially never mutated in UM. We have previously shown that UM frequently overexpress the p53 inhibitors mouse double minute (MDM)2 and/or MDMX^12^. Furthermore, we found that pharmacological activation of p53 or depletion of MDMX results in diminished UM cell growth and synergistically enhances DNA damage induced cell death^13^. Recently, it has been shown that the combination of an inhibitor of the MDM2–p53 interaction (CGM097^14^) with the broad PKC inhibitor Sotrastaurin did not achieve synergistic inhibition of cell growth in vitro^11^. Even so, in vivo four out of five PDX models showed a significant additive effect when AEB071 was combined with the MDM2 inhibitor CGM097.

In this study, we re-activated p53 by Nutlin-3 treatment and demonstrate that the combination of Nutlin-3 with Sotrastaurin does synergistically inhibit UM cell growth in vitro. Our data suggest these synergistic effects are due to a switch from a p53-induced cell cycle arrest to a pro-apoptotic response in combination with PKC inhibition. Detailed genetic studies showed that depletion of MDMX from UM cells enhances the efficacy of pan-PKC inhibition and, vice versa, PKCδ depletion sensitizes UM cells for p53 activation. Our results indicate that specifically targeting MDMX or PKCδ are potential new avenues for effectively treating UM patients in combination with PKC inhibitor(s) or p53 reactivation, respectively.

## Results

### Synergistic growth inhibition upon PKC inhibition and p53 reactivation

**Fig. 1:**
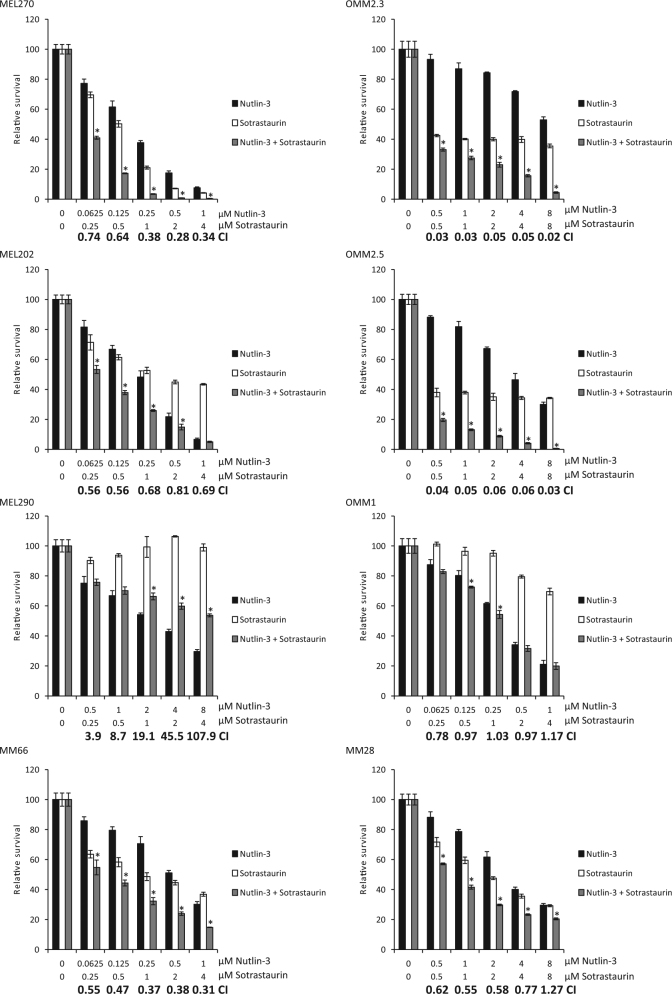
Synergistic growth inhibition by Sotrastaurin and Nutlin-3 in GNAQ/11 mutated UM cells. Various UM cell lines were treated for 72 h with indicated concentrations Sotrastaurin and Nutlin-3 alone or in combination to determine the effect on cell viability. Data plotted are the normalized averages with the standard deviation as error bars. To determine putative synergism, the combination index (CI) values were calculated with the Compusyn software. CI values below 0.9 were considered to be synergistic, between 0.9 and 1.1 additive and above 1.1 to be antagonistic. Combinations, which survival significantly differed compared with both single treatments, are indicated with an asterisk (*)

We first examined whether p53 reactivation (Nutlin-3) in combination with PKC inhibition (Sotrastaurin) synergistically inhibits the growth of UM cells (Fig. 1). In cell lines MEL270, MEL202, MM66, OMM2.5, OMM2.3 and MM28, combining Sotrastaurin with Nutlin-3 resulted in synergistic growth inhibition. So far the OMM1 cell line is the only exception of all GNAQ/11 mutated cell lines tested in which Sotrastaurin does not significantly enhance the Nutlin-3 effect. As expected, and shown before^9^, MEL290 cells, lacking a GNAQ/11 mutation, are not responsive to Sotrastaurin and the combination of Nutlin-3 and Sotrastaurin is even antagonistic.

### Combined Nutlin-3 and Sotrastaurin treatment promotes apoptosis

**Fig. 2:**
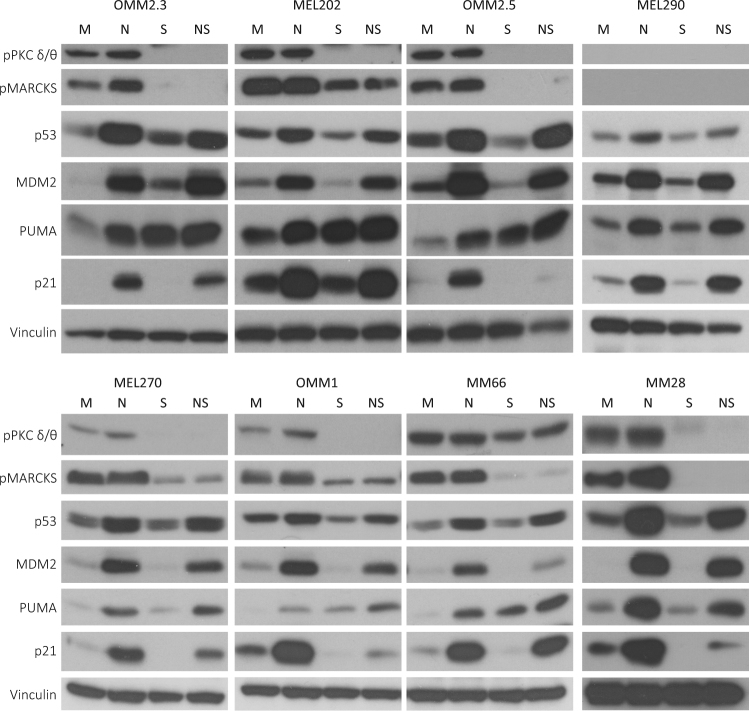
Biochemical response of uveal melanoma cell lines to Sotrastaurin and Nutlin-3. Cell lines OMM2.3, OMM2.5 and OMM1 were treated with 8 µM Nutlin-3 and 4 µM Sotrastaurin. MEL290 was incubated with 2 µM Nutlin-3 and 4 µM Sotrastaurin, cell line MM28 with 8 µM Nutlin-3 and 1 µM Sotrastaurin, and cell lines MEL202, MEL270 and MM66 with 2 µM Nutlin-3 and 0.5 µM Sotrastaurin. All cell lines were incubated for 24 h after which cells were harvested. Protein lysates were analyzed for the expression levels of phosphorylated PCKδ/θ, phosphorylated MARCKS, p53, MDM2, PUMA, p21 by western blot. Expression of vinculin was analyzed to control for equal loading

To assess target engagement of Sotrastaurin in the GNAQ/11 mutated UM cell lines, we determined the levels of phosphorylated PKCδ/θ and phosphorylated myristoylated alanine-rich c-kinase substrate (MARKS) (Fig. 2). The levels of these phosphorylated proteins almost disappeared or were significantly reduced upon Sotrastaurin treatment, confirming inhibition of the PKC activity. In GNAQ/11 wild-type cell line, MEL290 neither phosphorylated PKCδ/θ nor phospho-MARCKS could not be detected, as has previously been reported^9^. Effectivity of Sotrastaurin was further confirmed by the reduced mRNA levels of *CDC25A*, *Survivin* and *Cyclin D1* in the treated cells (Supplementary Figure 1S), as has been reported before^8,15^. Interestingly, in most cell lines Sotrastaurin also increased the levels of the pro-apoptotic protein PUMA. Treating cells with Nutlin-3 resulted in increased levels of p53 protein in all cell lines, with a concomitant increase in expression of known target genes/proteins (e.g., p21, MDM2 and PUMA; Fig. 2, Supplementary figure 1S). Furthermore, p53 reactivation repressed the expression of the pro-survival gene *Survivin* (Supplementary Figure 1S).

Combined Sotrastaurin/Nutlin-3 treatment slightly further increased the levels of the pro-apoptotic PUMA protein compared with single treatments while, in contrast, *CDC25A* and *Survivin* mRNA levels and p21 protein were reduced in most cell lines compared with single treatments. The mRNA levels of p21 were not reduced upon combinatory treatment, suggesting that the p21 protein reduction is regulated at a post transcriptional level. These results suggest that the pro-apoptotic response remains the same or is slightly increased in the combination treatment, but that the cell cycle arrest and pro-survival response is reduced, indicative of a shift from a cell cycle arrest to apoptosis.

**Fig. 3:**
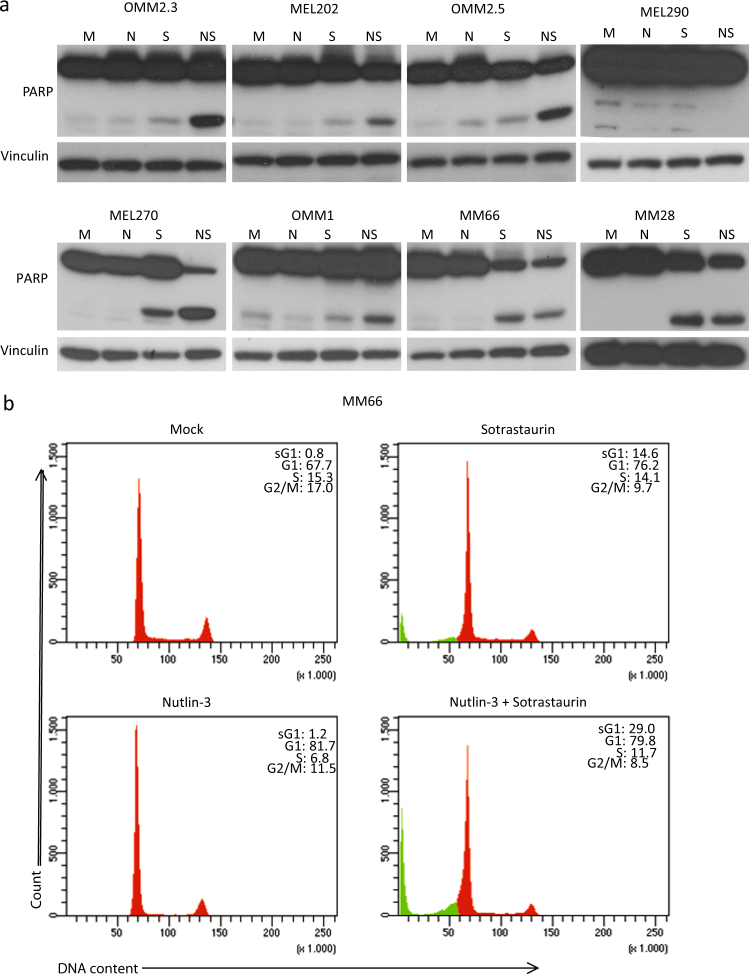
Induction of apoptosis upon combined p53 activation with PKC inhibition. **a** Cell lines OMM2.3, OMM2.5 and OMM1 were treated with 8 µM Nutlin-3 and 4 µM Sotrastaurin. MEL290 was incubated with 2 µM Nutlin-3 and 4 µM Sotrastaurin, cell line MM28 with 8 µM Nutlin-3 and 1 µM Sotrastaurin. and cell lines MEL202, MEL270 and MM66 with 2 µM Nutlin-3 and 0.5 µM Sotrastaurin. All cell lines were incubated for 72 h before harvesting. Protein lysates were analyzed for the expression levels of cleaved and full-length PARP by western blot. Expression of vinculin was analyzed to control for equal loading. **b** MM66 cells were incubated with 2 µM Nutlin-3 and 0.5 µM Sotrastaurin for 72 h after which the cell cycle profiles were determined by flow cytometry after PI staining, showing an increase in the subG1 fraction upon combined treatment

To study whether the observed shift in biochemical response results in increased apoptosis, we investigated poly (ADP-ribose) polymerase (PARP) cleavage as a marker for apoptosis because upon induction of apoptosis, the PARP protein gets cleaved by activated caspases. Clear increased levels of cleaved PARP were detected in cell lines MEL270, OMM2.3 and OMM2.5 when treated with Nutlin-3/Sotrastaurin (Fig. 3a) and to a lesser extent in OMM1 and MEL202 cells. In MM66 and MM28 cells, Sotrastaurin treatment alone already resulted in PARP cleavage, which was not further enhanced by addition of Nutlin-3 (Fig. 3a). However, in MM66 and MM28 the full-length PARP levels in the combined treated cells decreased, indicating that the percentage of cleaved PARP compared with full-length still increased in the Nutlin-3/Sotrastaurin-treated cells. Additionally, cleaved caspase 3 was increased in MM66 and MM28 cells further indicating the induction of apoptosis (Supplementary Figure 2a). No PARP cleavage was observed in MEL290 cells.

As the main goal is to find better treatment for UM metastases, further experiments have been performed with metastasis-derived cell lines only. Flow cytometry analysis was performed to examine the effects of the drugs on the cell cycle progression and a possible induction of a subG1 fraction, indicative of cell death. The MM66 and OMM1 cells showed an arrest in the G1 phase upon p53 reactivation. This effect was not obvious in MM28 cells, possibly also because these cells grow very slowly and the population of untreated cells already contains 87% of cells in G1 phase (Fig. 3b and Supplementary Figure 2Sb). Nutlin-3 treatment did slightly increase subG1 fraction in MM28 cells. Cell cycle profiles of OMM2.3 and OMM2.5 cells were only slightly affected by treatment with Nutlin-3. Sotrastaurin treatment induced an accumulation of cells in G1 phase in all cell lines. In MM66 and MM28 cell lines, Sotrastaurin also increased cells in subG1 (14.6 and 21.0%, respectively), in concordance with the analysis of PARP cleavage. Combining Nutlin-3 and Sotrastaurin slightly increased number of G1 cells in OMM2.5 and OMM2.3, but not in the other cell lines. Importantly, simultaneous p53 reactivation and PKC inhibition significantly increased the fraction of subG1 cells in all cell lines, most strikingly in MM28, MM66 and OMM2.5 cells. In conclusion, these results together with the PARP cleavage analysis indicate that the combination of Nutlin-3 and Sotrastaurin is more potent in the induction of apoptosis compared with the single treatments.

### MDMX depletion enhances growth inhibitory effect of Sotrastaurin

**Fig. 4:**
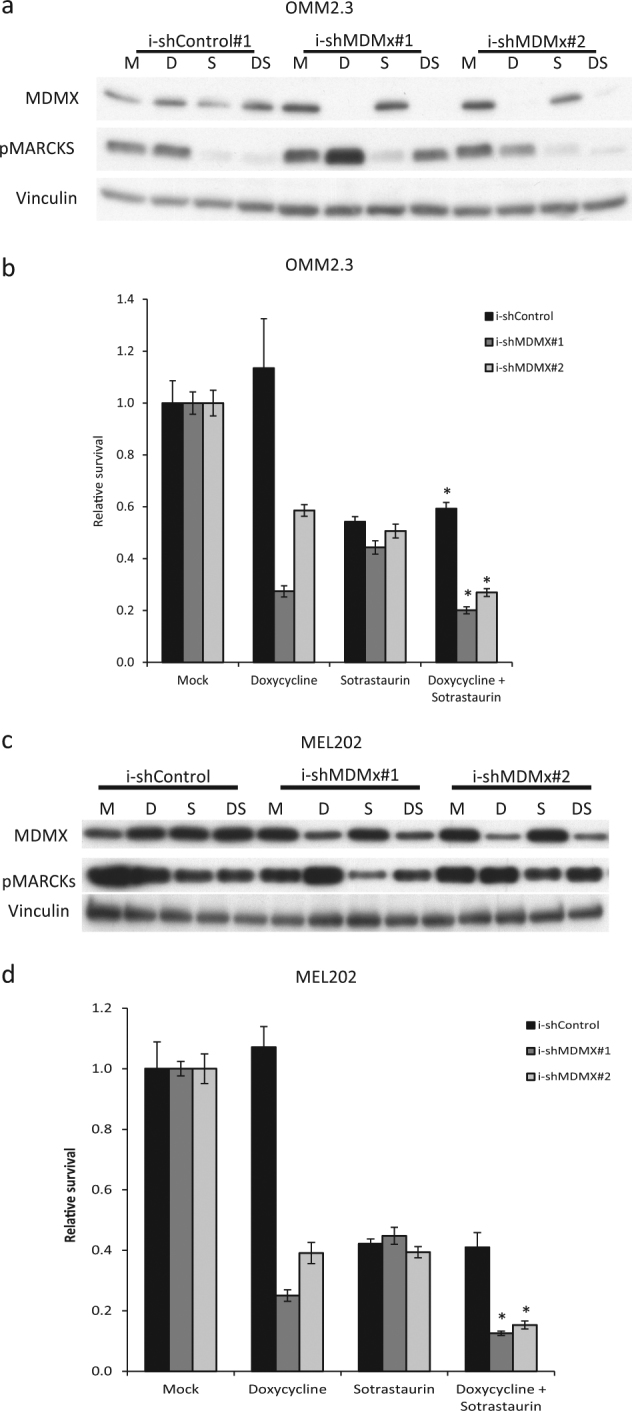
MDMX depletion inhibits UM cell growth and increases growth inhibition by Sotrastaurin treatment. **a**, **c** OMM2.3 and MEL202 i-shCtrl and i-shMDMX cells were incubated for 72 h with 10 ng/ml doxycycline, 0.5 µM Sotrastaurin or the combination before harvesting. The expression of MDMX and phosphorylated MARCKS was analyzed by western blot. Vinculin expression was analyzed to control for equal loading. **b**, **d** OMM2.3 and MEL202 i-shCtrl and i-shMDMX cells were seeded in quadruplicate in 12-well plates and incubated for 8 days with indicated compounds (OMM2.3: 20 ng/ml doxycycline and 0.5 µM Sotrastaurin; MEL202: 20 ng/ml doxycycline and 0.1 µM Sotrastaurin). Cell survival was determined using crystal violet staining. Data plotted are the normalized averages with the standard deviation as error bars. Combinations, which survival significantly differed compared with both single treatments, are indicated with an asterisk (*)

As ‘specific’ MDM2 inhibitors in the clinic have shown strong adverse effects^16^, we determined whether specific targeting of MDMX could serve as an alternative for MDM2 inhibitor-based therapies in UMs, especially in combination with Sotrastaurin. Therefore, we created OMM2.3- and MEL202-derived cell lines containing two distinct MDMX-targeting short hairpin RNAs (shRNAs; i-shMDMX) or control shRNA (i-shCtrl) under control of a doxycycline-inducible promoter. Inducing shRNA expression with doxycycline resulted in depletion of MDMX protein in the i-shMDMX containing cells with no effect in the i-shCtrl cells (Fig. 4a, c). Concomitantly, depletion of MDMX activated p53 signaling with upregulation of mRNA levels of p53 target genes *MDM2*, *CYFIP2*, *MAD2L1* and *KIF23* in MEL202 and *p21* in both OMM2.3 and MEL202 (Supplementary Figure 3Sa and b). Although OMM2.3 cells express rather low basal levels of MDMX protein, depletion of MDMX still resulted in growth inhibition (38–53% survival) in a long-term growth assay (Fig. 4b). Growth inhibition upon Sotrastaurin treatment was comparable in the OMM2.3-derived cell lines (~55% survival). Adding Sotrastaurin to MDMX-depleted cells further reduced cell survival to 21–28% (Fig. 4b). MEL202 cells showed a 45–47% survival upon MDMX depletion and a 46–60% survival upon PKC inhibition. Combining MDMX depletion with PKC inhibition resulted in a further reduction of survival of ~30%, an reduction of ~20% compared with Sotrastaurin (Fig. 4c, d). However, in both cell lines, the Excess over Bliss (EoB) scores did not suggest synergism. These results suggest that targeting MDMX could be further explored as an alternative for p53 activation by MDM2 inhibitors in obtaining enhanced UM growth inhibition by PKC inhibition with Sotrastaurin.

### PKCδ depletion sensitizes UM cells for p53 activation

**Fig. 5:**
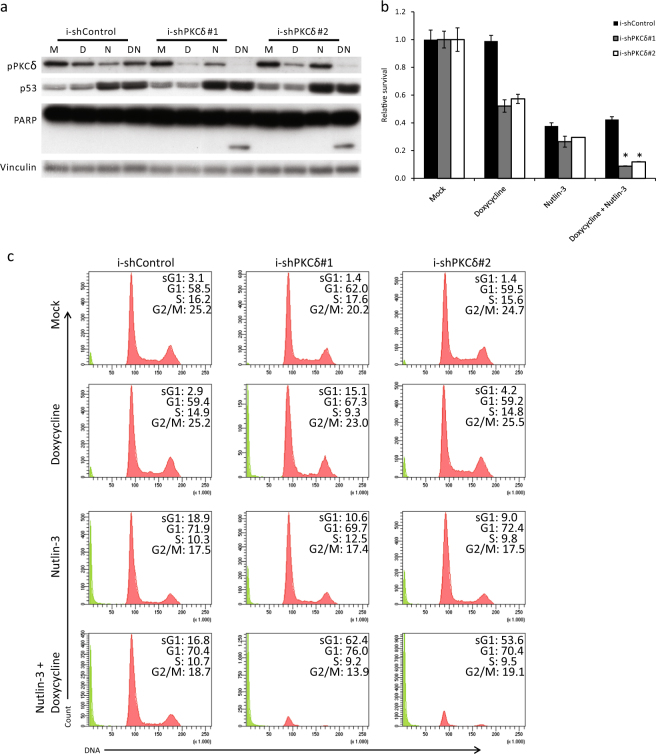
PKCδ depletion and Nutlin-3 synergize to induce apoptosis in UM cells. **a** OMM2.5 i-shCtrl and i-shPKCδ cells were incubated for 72 h with 20 ng/ml doxycycline, 8 µM Nutlin-3 or the combination of compounds. Expression of phosphorylated PKCδ, p53 and PARP was determined using western blot. Expression of vinculin was assessed to control for equal loading. **b** OMM2.5 i-shCtrl and i-shPKCδ cells were seeded into 96-well plates and incubated with 20 ng/ml doxycycline and 4 µM Nutlin-3. After 5 days of incubation, cell survival was determined by CTB measurement. Data plotted are the normalized averages with the standard deviation as error bars. Combinations, which survival significantly differed compared with both single treatments, are indicated with an asterisk (*). **c** Cell cycle profiles of OMM2.5 i-shCtrl and i-shPKCδ cells were determined after 72 h of treatment with indicated drugs by flow cytometry after PI staining, showing an increase in the subG1 fraction upon combined treatment

Previous studies have shown non-redundant and essential roles for various PKC isoforms in cancer cell growth. One of the PKC isoforms shown to be essential for UM cell growth and proliferation is PKCδ^8,17^. To specifically target PKCδ in MEL202 and OMM2.5 cells, we introduced lentiviral constructs which inducible express PKCδ-targeting shRNAs (i-shPKCδ; two distinct target sequences) or control shRNA (i-shCtrl). Incubating the cells with doxycycline strongly reduced PKCδ levels in the i-shPKCδ cells, without effecting PKC isoforms α, β and ζ/λ. Doxycycline has no effect on PKCδ levels in the i-shCtrl cells (Fig. 5a and Supplementary Figure 4Sa and c). Depletion of PKCδ reduced OMM2.5 cell survival to 52–57% (Fig. 5b). OMM2.5 cells expressing i-shCtrl or i-shPKCδ shRNAs showed similar sensitivity to Nutlin-3 treatment with a survival of 29–38%. Combining PKCδ depletion with Nutlin-3 reduced cell survival to 9–12%, a reduction of ~17% for both inducible shRNA’s compared with Nutlin-3 alone, which results in high synergistic EoB values of 5.0 (Fig. 5b). Interestingly, PARP cleavage could only be detected in the Nutlin-3-treated cells depleted for PKCδ, indicating the triggering of apoptosis (Fig. 5a). Indeed, the induction of cell death was confirmed by flow cytometry analysis, which showed a strong increase in the fraction of subG1 cells in Doxycycline/Nutlin-3-treated i-shPKCδ cells, whereas single treatments mainly show a minor induction of subG1 and a G1 arrest (Fig. 5c). Synergism was also observed in MEL202 cells when PKCδ depletion was combined with Nutlin-3 in a long-term growth assay, with EoB scores of 5.5 and 9.4 (Supplementary Figure 4Sb). These data indicate that just PKCδ depletion is sufficient to replace pan-PKC inhibition for achieving synergist cell growth inhibitory effects in combination with p53 reactivation.

## Discussion

UM is considered to be a rare disease with an incidence of approximately 6 per million, accounting for 5% of all melanoma cases^18^. Melanomas originating from the uvea are most commonly driven by activating mutations in G-proteins GNAQ (50%) or GNA11 (43%)^4,5^. These distinct mutations, among additional effects, hyper-activate PKC isoforms, which in turn feed into the MAPK pathway. This insight has spurred the development of several (pan-) PKC inhibitors, including Sotrastaurin. Despite the effectivity of Sotrastaurin in vitro on the growth of UM cell lines, in a phase I clinical trial only modest effects as single therapy were observed^10^. To enhance the effect of the PKC inhibitor, a trial was started with Sotrastaurin in combination with a MEK inhibitor. Unfortunately, this clinical trial had to be terminated prematurely due to toxicity issues. The urge for novel therapeutic interventions has spiked the interested to combine PKC inhibition with compounds inspired by other key features of UM. One of these features is the lack of p53 mutations. UMs frequently show high levels of MDM2 and/or MDMX to constrain p53 tumor-suppressor activity, opening the possibility to use MDM2/X inhibitors such as Nutlin-3 to reactivate p53. Previous studies have already shown that MDM2/X inhibitors have the potential to be used as therapeutic intervention for UM^12,13,19^.

During the course of our studies, it has been reported that inhibition of p53 regulation by MDM2 using CGM097 further constrained in vivo tumor growth in UM PDX models when combined with PKC inhibition, although this combination did not result in synergistic growth inhibition in vitro^11^. In contrast, our results clearly show synergistic effects when p53 reactivation is combined with PKC inhibition with the same PKC inhibitor and also with an alternative inhibitor GF109203X (data not shown). This apparent controversy can possibly be explained by the use of distinct p53 reactivators. We have previously shown that Nutlin-3 not only prevents the MDM2/p53 complex, but also affects the MDMX–p53 interaction, which has not been investigated for the MDM2 inhibitor CGM097. It could suggest that only inhibiting the MDM2–p53 interaction might not be sufficient to fully unleash p53 and achieve a synergistic response, at least in vitro. Even so, functional MDM2 inhibition might not be the optimal way to go in patients, due to the previous reported adverse effects^16,20,21^. Therefore, we focused our studies on targeting MDMX, because mouse studies have indicated that depletion of MDMX has much less detrimental effects on the well-being of the organism, most likely because MDMX is less universal expressed in adult tissues. We demonstrate here that depletion of MDMX also enhanced the growth inhibitory effects of PKC inhibition. Furthermore, we have shown previously that MDMX has oncogenic effects beyond inhibition of p53^12,19,22,23^, so targeting MDMX might have more wide-ranging tumor growth inhibitory effects than merely p53 reactivation.

Unfortunately, to date, no small-molecule compound specifically targeting MDMX is commercially available. It had been reported that the XI-011 compound decreases MDMX levels in tumor cells by blocking transcription of the *MDMX* gene^24^. However, we have shown previously that this compound not only affects MDMX levels but also clearly elicits a DNA damage response, making the mode-of-action of this compound rather complex^12^. Much more promising, Dewaele and colleagues recently showed the potential of stimulating the naturally occurring alternative splicing of *MDMX* by antisense oligonucleotides, thereby decreasing the amount of full-length MDMX protein^25^. The depletion of MDMX resulted in inhibition of cutaneous melanoma growth, both in vitro and in PDX mouse models. These results combined with ours strongly suggest a potential therapeutic intervention to target metastasized UM.

Previous studies have demonstrated the non-redundant and often essential roles of different PKC isoforms in UM^8,17^. We sought to determine whether an inhibition or depletion of a single PKC isoform could also be capable of enhancing MDM2/MDMX inhibition. Xinqi Wu and colleagues showed in 2012 in two independent studies that PKC isoforms α, β, θ, ε and δ are essential for UM cell line viability^8,17^. More recently, it has been reported that both PKCε and PKCδ are needed for the activation of RASGRP3 driving the RAS/MEK/ERK pathway^26^. Confirming these studies, we show that UM cell viability depends on PKCδ and therefore could provide a potential drug target, especially as PKCδ does not seem to be required for development and normal cell proliferation^27,28^. Interestingly, the depletion of a single PKC isoform not only reduced cell viability, but also synergistically enhanced the effects of Nutlin-3. Our data show that this reduced cell survival is due to the induction of cell death, most likely via apoptosis, providing an interesting therapeutic potential. In conclusion, the combination of an isoform-selective PKC inhibitor with MDMX inhibition might be a new potent therapeutic intervention for UM metastases with limited adverse effects.

## Methods

### Cell culture and lentiviral transduction

Cell lines MEL270, MEL202, OMM2.3, MEL290, OMM2.5 and OMM1 were cultured in a mixture of RPMI and Dulbecco’s modified Eagle’s medium F12 (1:1 ratio), supplemented with 10% fetal calf serum (FCS) and antibiotics. MM66 and MM28 were cultured in Iscoves's modified Dulbecco's medium containing 20% FCS and antibiotics. Inducible shRNA knockdown lentiviral vectors were constructed as described previously^29,30^. Production of lentivirus stocks by transfections into 293T cells essentially as described, but calcium phosphate was replaced with polyethylenimine^31^. Virus was quantitated by antigen capture enzyme-linked immunosorbent assay (ELISA) measuring HIV p24 levels (ZeptoMetrix Corp., New York, NY, USA). Cells were transduced using a multiplicity of infection of 2 in medium containing 8 μg/ml polybrene. Target sequences to deplete MDMX or PKCδ and control sequences are shown in Table 1. All cells were frequently tested for mycoplasma infection.

**Table 1:** shRNA target sequences

Target	shRNA sequence
Control	5′-GAATCTTGTTACATCAGCT-3'
PKCδ#1	5′-CAGAGCCTGTTGGGATATATC-3'
PKCδ#2	5′-CTTCGGAGGGAAATTGTAAAT-3'
MDMX#1	5′-GTGCAGAGGAAAGTTCCAC-3'
MDMX#2	5′-GAATCTCTTGAAGCCATGT-3'

### Western blot analysis

Cells were washed twice in ice-cold phosphate-buffered saline (PBS) and lysed in Giordano buffer (50 mM Tris-HCl pH7.4, 250 mM NaCl, 0.1% Triton X-100 and 5 mM EDTA; supplemented with phosphatase and protease inhibitors). Equal protein amounts were separated using sodium dodecyl sulfate–polyacrylamide gel electrophoresis and blotted on polyvinylidene fluoride transfer membranes (Millipore, Darmstadt, Germany). After blocking the membranes in Tris-bufferd saline and tween 20 (10 mM Tris-HCl pH8.0, 150 mM NaCl, 0.2% Tween-20) containing 10% non-fat dry milk, membranes were incubated with the proper primary antibodies (listed in a Table 2) and appropriate horseradish peroxidase-conjugated secondary antibodies (Jackson Laboratories, Bar Harbor, MA, USA). Bands were visualized using chemoluminescence and autoradiography.

**Table 2:** List of antibodies used for western blot

Protein	Name/cat#	Company
MDM2	SMP14/sc-965	Santa Cruz Biotechnology
MDM2	3G9/04-1530	Millipore
MDMX	8C6/04-1555	Millipore
p21	CP74/05-655	Millipore
p53	DO1/sc-126	Santa Cruz Biotechnology
PARP	9542	Cell Signaling Technology
pMARCKS	2741	Cell Signaling Technology
PKCδ	EPR17075/ab182126	Abcam
pPKCα/β	9375	Cell Signaling Technology
pPKCβ	9371	Cell Signaling Technology
pPKCδ	9374	Cell Signaling Technology
pPKCδ/θ	9376	Cell Signaling Technology
pPKCζ/λ	9378	Cell Signaling Technology
PUMA	G3/sc-374223	Santa Cruz Biotechnology
Vinculin	hVIN-1/V9131	Sigma-Aldrich

### RNA isolation, cDNA synthesis and real-time quantitative PCR

RNA was isolated from cells using the SV total RNA isolation kit (Promega, Fitchburg, WI, USA), from which complementary DNA (cDNA) was synthesized using the reverse transcriptase reaction mixture as indicated by Promega. Quantitative PCR (qPCR) was performed using SYBR green mix (Roche, Basel, Switzerland) in a C1000 touch Thermal Cycler (Bio-Rad Laboratories, Hercules, CA, USA). Relative expression of *CDC25A*, *cyclin D1*, *survivin*, *p21* and *MDM2* was determined over three independent experiments, compared with housekeeping genes *CAPNS1* and *SRPR*. Relative expression levels per experiment were compared and the untreated samples average was set at 1. Primer sequences are listed in Table 3.

**Table 3:** Sequences of qPCR primers

Name	Sequence
CDC25A fw	5′-CTCCGAGTCAACAGATTCAGG-3'
CDC25A rev	5′-TTCAAGGTTTTCTTTACTGTCCAA-3'
Cyclin D1 fw	5′-GAAGATCGTCGCCACCTG-3'
Cyclin D1 rev	5′-GACCTCCTCCTCGCACTTCT-3'
Survivin fw	5′- GCCCTTTCTCAAGGACCA-3'
Survivin rev	5′-CAGCTCCTTGAAGCAGAAGAA-3'
p21 fw	5′-AGCAGAGGAAGACCATGTGGA-3'
p21 rev	5′-AATCTGTCATGCTGGTCTGCC-3'
MDM2 fw	5′-ACGCACGCCACTTTTTCTCT-3'
MDM2 rev	5′-TCCGAAGCTGGAATCTGTGAG-3'
CAPNS1 fw	5′-ATGGTTTTGGCATTGACACATG-3'
CAPNS1 rev	5′-GCTTGCCTGTGGTGTCGC-3'
SRPR rw	5′-CATTGCTTTTGCACGTAACCAA-3'
SRPR rev	5′-ATTGTCTTGCATGCGGCC-3'
CYFIP2 fw	5′-CAGCCCAACCGAGTAGAGAT-3'
CYFIP2 rev	5′-CTTCACCTCGCTGCAGAAC-3'
KIF23 fw	5′-TGCTGCCATGAAGTCAGCGAGAG-3'
KIF23 rev	5′-CCAGTGGGCGCACCCTACAG-3'
MAD2L1 fw	5′-AAGTGGTGAGGTCCTGGAAA-3'
MAD2L1 rev	5′-TTCCAACAGTGGCAGAAATG-3'

### Flow cytometry analysis

To analyze cell cycle profiles, the cells were harvested using trypsinization, washed with ice-cold PBS and fixed in ice-cold 70% ethanol. Cells were washed in PBS containing 2% FCS and resuspended in PBS containing 2% FCS, 50 µg/ml RNAse and 50 µg/ml propidium iodide. Flow cytometry was performed using the BD LSR II system (BD Bioscience, San Diego, CA, USA). In all, 10,000 cycling cells were analyzed and percentages G1, S and G2/M were determined and set to 100%. The subG1 population was determined as a percentage of the total population.

### Cell growth and viability assays

Cells were seeded in triplicate, in 96-well format. Next day, compounds were added and cells were incubated for 72 h. Cell survival was determined using CellTitre-Blue Cell Viability assay (Promega); fluorescence was measured in a microplate reader (Victor3, Perkin Elmer, San Jose, CA, USA). Synergism between Sotrastaurin and Nutlin-3 was calculated using Compusyn software (Paramus, NJ, USA). Sotrastaurin and Nutlin-3 were obtained from Selleck Chemicals (Houston, TX, USA) and Cayman Chemical (Ann Arbor, MI, USA), respectively.

### Long-term growth assay

Cells were seeded in triplicate in a 12-well plates and were incubated for 8 days. Cells were fixed for 5 min in 4% paraformaldehyde. DNA was stained using 30-min incubation with 0.05% crystal violet. After washing and drying, the relative number of cells was quantified by solubilizing the crystal violet in 100% methanol and measuring absorbance at 545 nM using a microplate reader (Victor3, Perkin Elmer).

### Determining synergism

To determine the extent of synergism between Sotrastaurin and Nutlin-3 in UM cell lines, combination index (CI) values were calculated by comparing ranges of both single drugs (concentrations as indicated in the figure) to the combined treatment. Therefore, we used the CompuSyn program, which uses the Chou-Talalay method^32^. CI values below 0.9 were considered to be synergistic, between 0.9 and 1.1 additive effects and above 1.1 to be antagonistic. EoB was used to determine the synergism between two conditions as described by Amirouchene-Angelozzi et al.^33^ when two ranges of drugs were tested. EoB was used to determine the extent of synergism between MDMX depletion and Sotrastaurin and PKCδ depletion and Nutlin-3.

### Statistical analysis

Differences between two groups were calculated using Student’s *t*-test, two sided; *P*-values of 0.05 or less were considered to be significant. Variation between groups compared was found to be similar, using F-testing. Data shown are averages with ± S.D.

## Electronic supplementary material

Supplementary figure legends

Supplementary Figure 1

Supplementary Figure 2

Supplementary Figure 3

Supplementary Figure 4
