# Supplementary material for: Targeting MDMX and PKCδ to improve current uveal melanoma therapeutic strategies
Source: Oncogenesis. 2018 Mar 29;7(3):33. doi: 10.1038/s41389-018-0041-y (PMC5874255; doi:10.1038/s41389-018-0041-y)
Supplement: Supplementary file 1 — Supplementary figure legends [file 41389_2018_41_MOESM1_ESM.docx]

**Supplementary figure legends**

**Supplementary Figure 1S. Analysis of gene transcription in response to Sotrastaurin and Nutlin-3.**

Cell lines OMM1, OMM2.5 (8 µM Nutlin-3 and 4 µM Sotrastaurin) and MM66 (2 µM Nutlin-3 and 0.5 µM Sotrastaurin) were incubated with Sotrastaurin and Nutlin-3 for 24 hours. Cells were harvested, RNA isolated, cDNA synthesized and expression of CDC25A, cyclin D1, survivin, p21 and MDM2 was determined. Relative expression compared to untreated controls is plotted.

**Supplementary Figure 2S. Induction of cell cycle arrest and cell death upon p53 activation and PKC inhibition.**

(**a**) MM66 and MM28 were incubated for 72 hours with Sotrastaurin (MM66: 4 µM and MM28 1 µM), 8 µM Nutlin-3 or the combination. Expression of cleaved caspase 3 was determined by Western blot. Expression of UPS7 was assessed to control for equal loading. (**b**) After treating OMM2.5, OMM1, OMM2.3 (8 μM Nutlin-3 and 4 μM Sotrastaurin) and MM28 (8 μM Nutlin-3 and 1 μM Sotrastaurin) for 72 hours the cell cycle profiles were determined with flow cytometry using PI staining. Representative figures of three independent experiments with the percentage of each cell cycle phase (G1, S, G2/M and subG1) are shown.

**Supplementary Figure 3S. p21 expression in response to MDMX knockdown**

(**a**) OMM2.3- and MEL202 i-shCtrl and i-shMDMX cells were incubated with 20 ng/ml doxycycline or solvent for 72 hours. Cells were harvested, RNA isolated, cDNA synthesized and expression levels of p21 mRNA was determined by qPCR. Relative expression compared to untreated is plotted. (**b**) Expression levels of known p53 target genes (MDM2, CYFIP2, MAD2L1 and KIF23) upon MDMX depletion in MEL202.

**Supplementary Figure 4S. PKCδ depletion sensitizes MEL202 cells for Nutlin-3.**

(**a**) MEL202 i-shCtrl and -i-shPKCδ cells were incubated for 72 hours with 20 ng/ml doxycycline, 1 µM Nutlin-3 or the combination. Expression of phosphorylated PKCδ, p53 and PARP was determined by Western blot. Expression of vinculin was assessed to control for equal loading. (**b**) MEL202 i-shCtrl and -i-shPKCδ cells were seeded in quadruplicate into 12-well plates and incubated for 8 days with 20 ng/ml doxycycline, 1 µM Nutlin-3 or the combination. Cell survival was determined using crystal violet staining. Data plotted are the normalized averages with the standard deviation as error bars. Combinations which survival significantly differed compared to both single treatments are indicated with an asterisk (*). (**c**) MEL202 i-shCtrl and -i-shPKCδ cells were incubated for 72 hours with 20 ng/ml doxycycline. Expression of PKCδ, phosphorylated PKC α, β, λ, and ξ was determined by Western blot. Expression of USP7 was assessed to control for equal loading.
