# Supplementary figures and images for: Targeting MDMX and PKCδ to improve current uveal melanoma therapeutic strategies
Source: Oncogenesis. 2018 Mar 29;7(3):33. doi: 10.1038/s41389-018-0041-y (PMC5874255; doi:10.1038/s41389-018-0041-y)

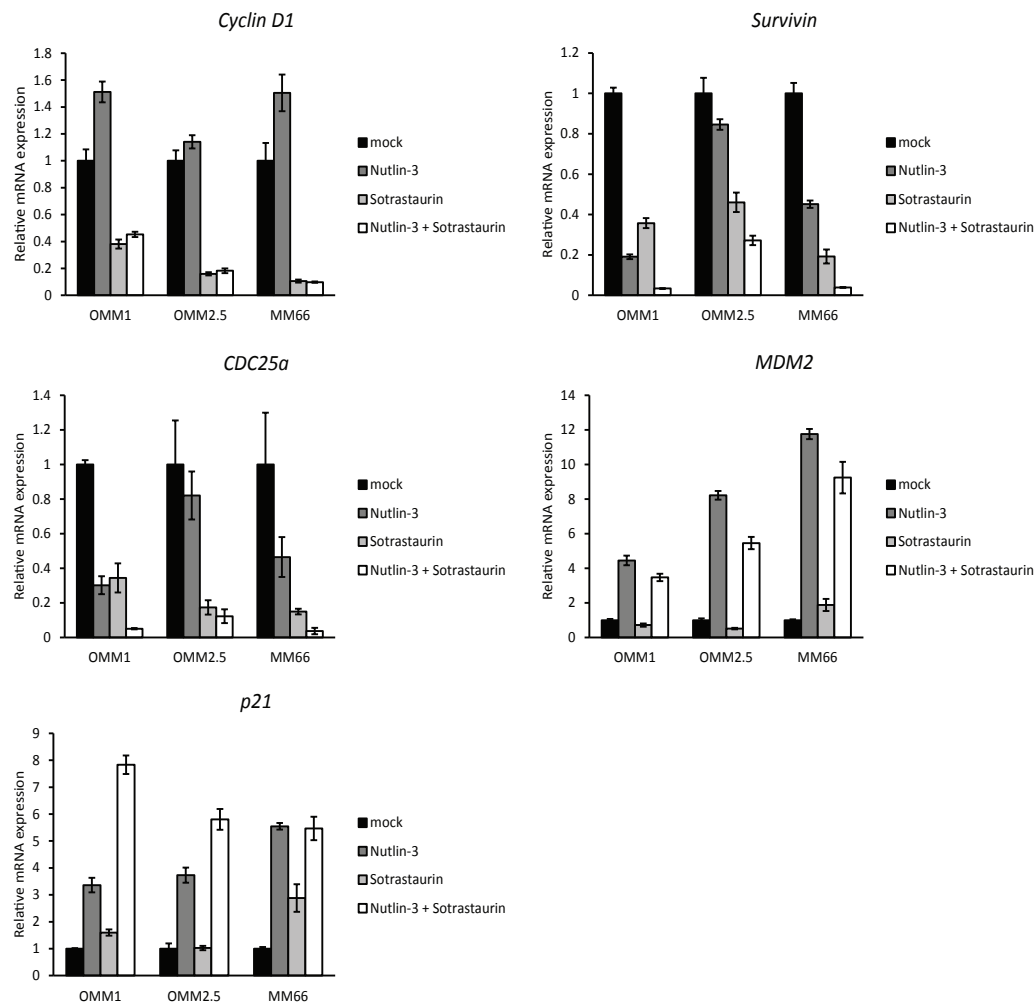

Supplement: Supplementary file 2 — Supplementary Figure 1 [file 41389_2018_41_MOESM2_ESM.pdf]

2S

a

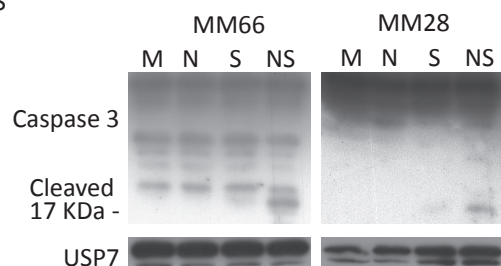

b

OMM2.5

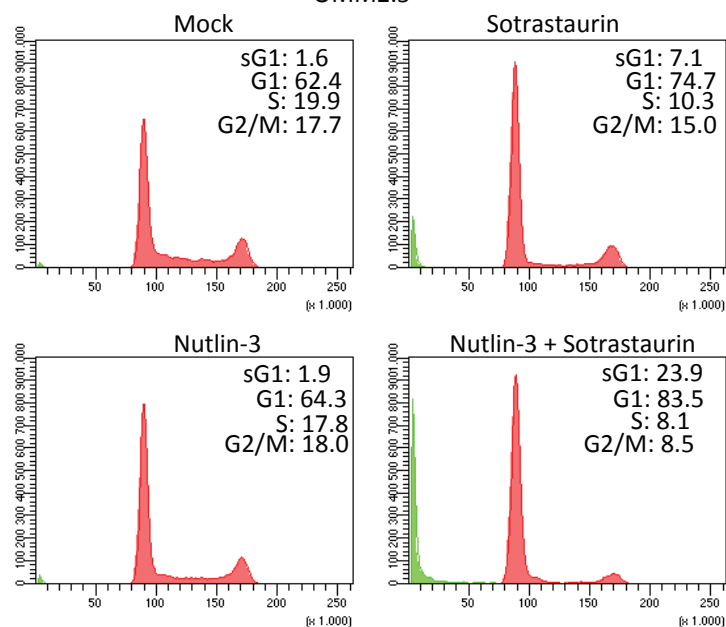

MM28

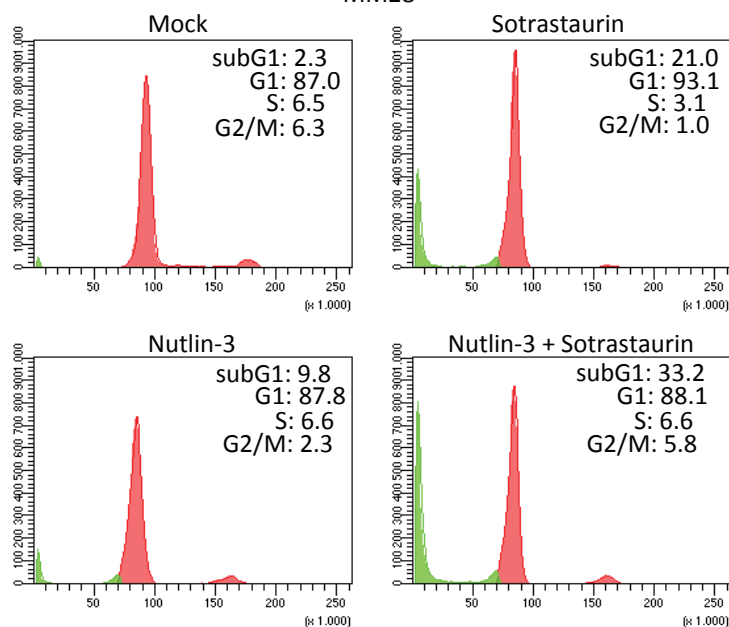

OMM2.3

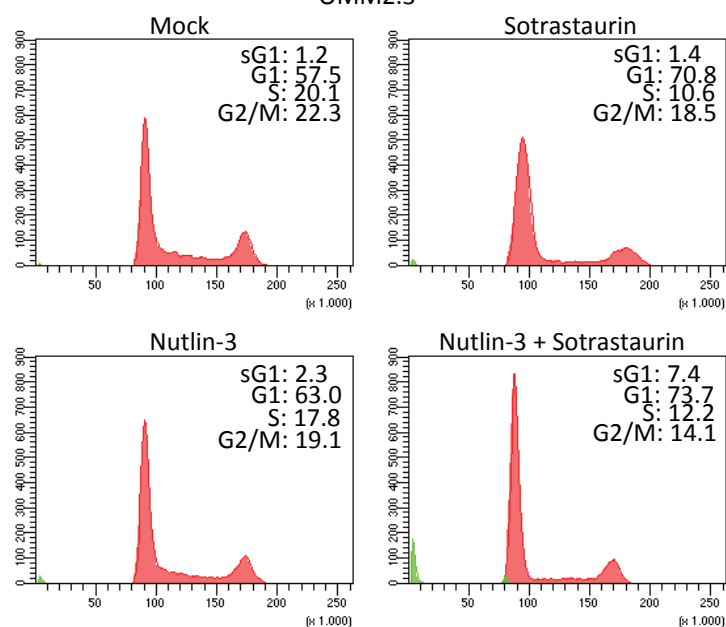

OMM1

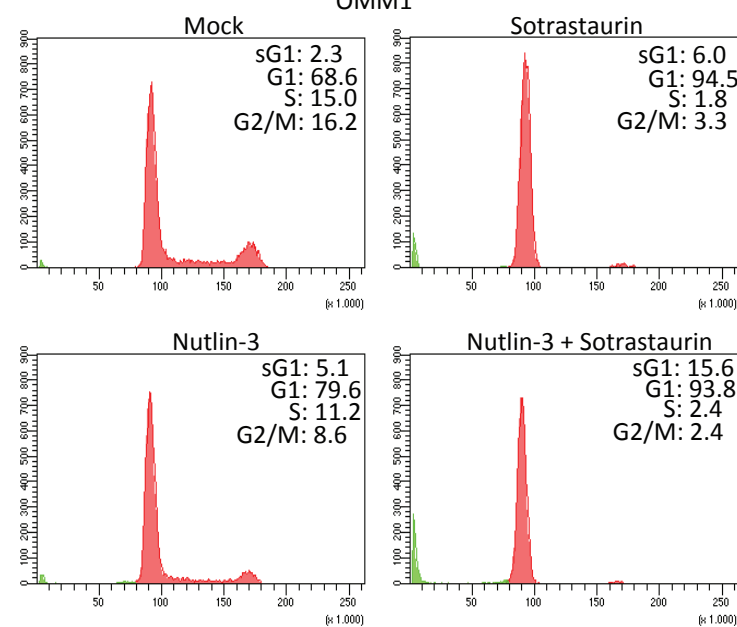

Supplement: Supplementary file 3 — Supplementary Figure 2 [file 41389_2018_41_MOESM3_ESM.pdf]

a

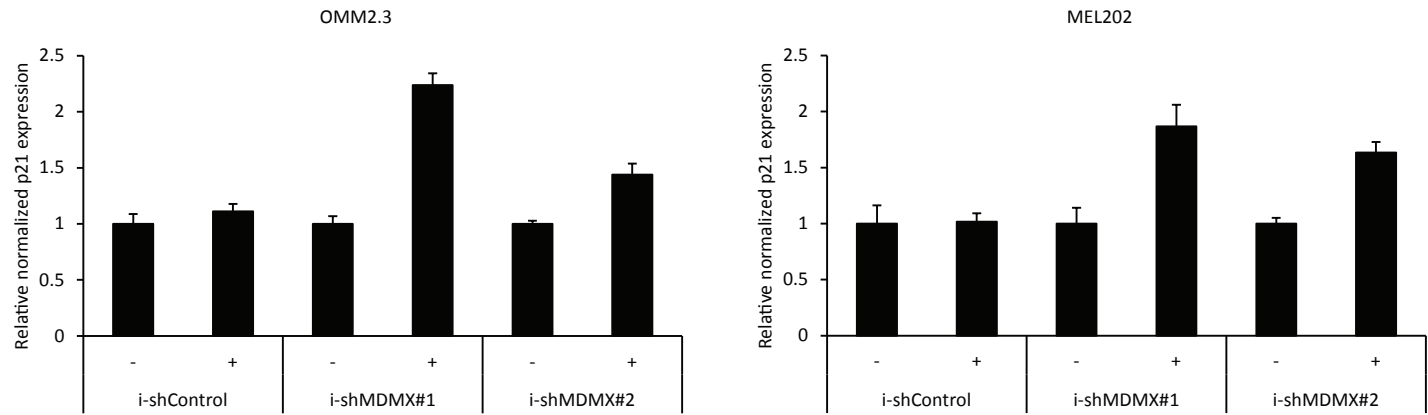

b

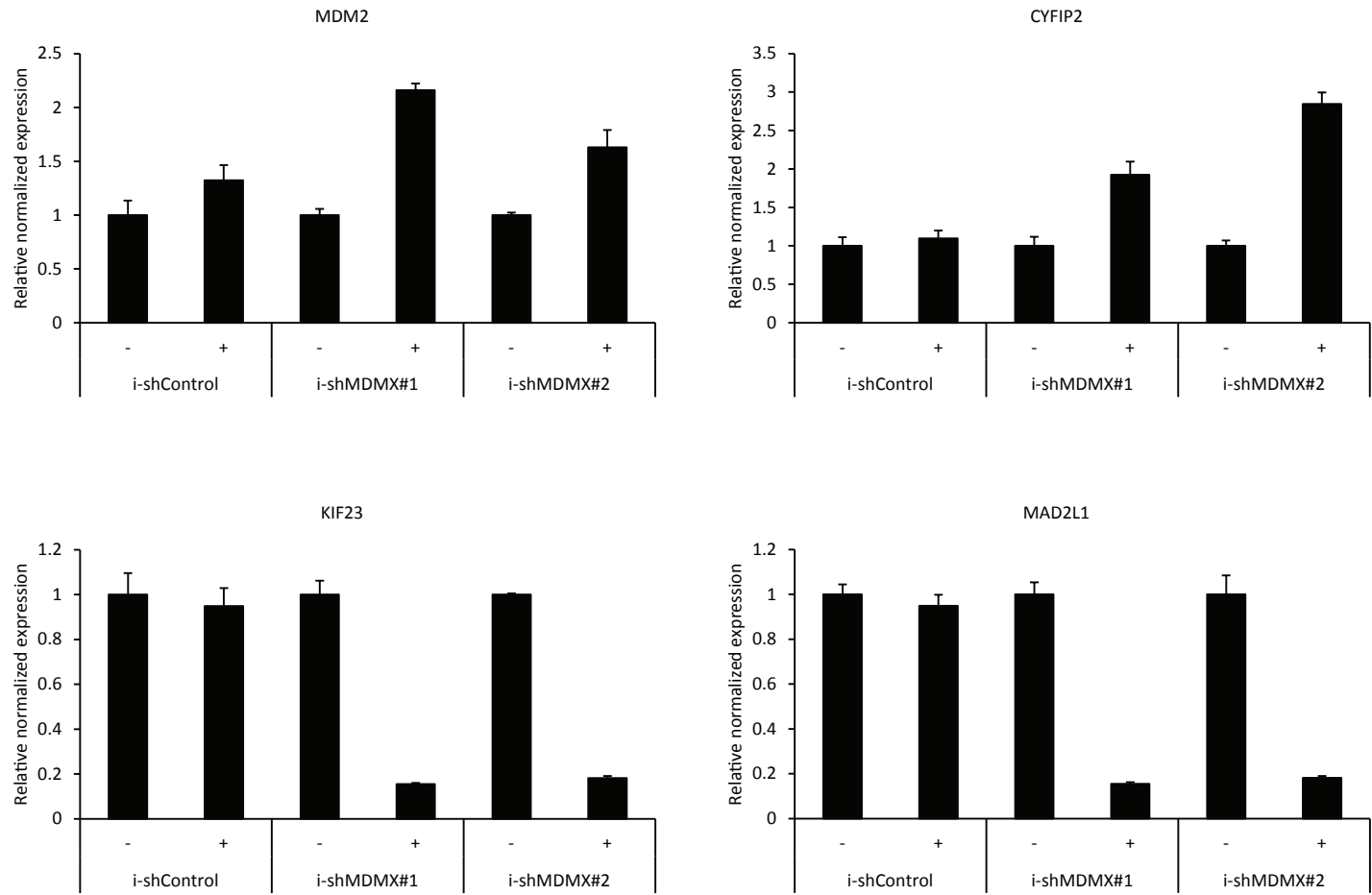

Supplement: Supplementary file 4 — Supplementary Figure 3 [file 41389_2018_41_MOESM4_ESM.pdf]

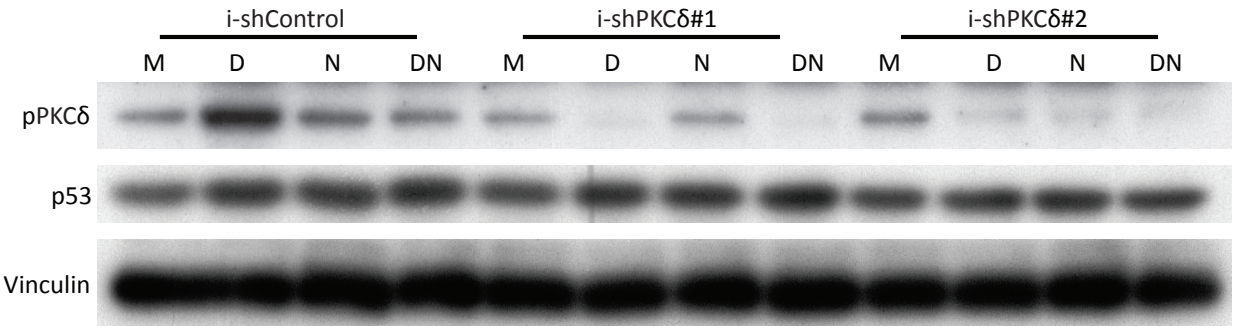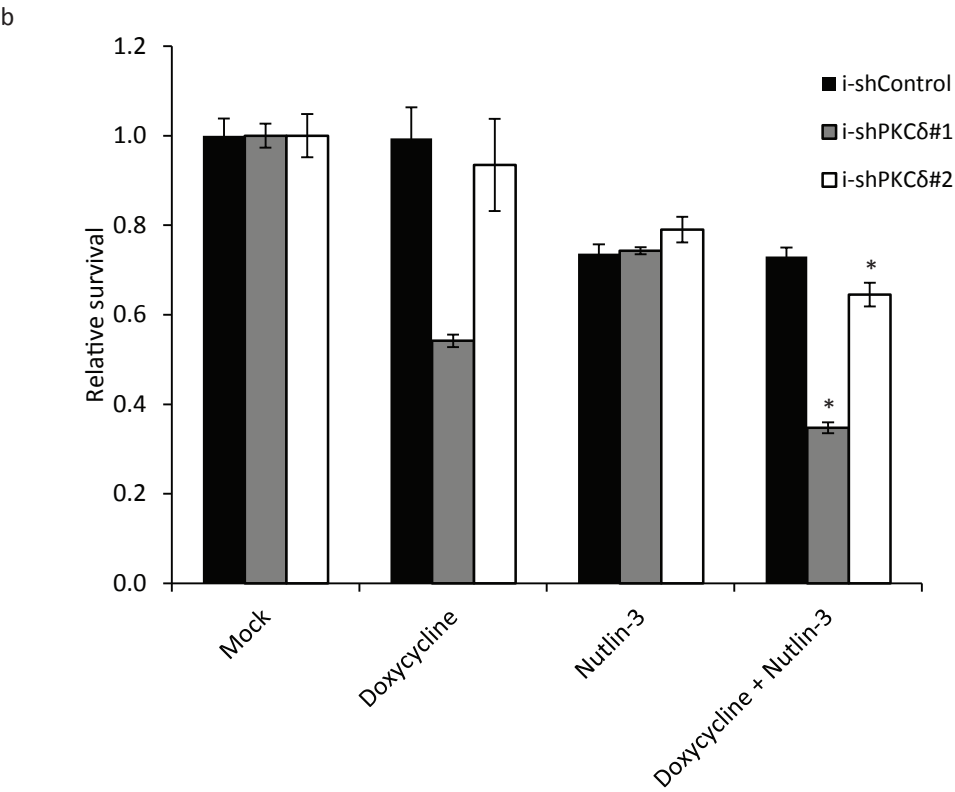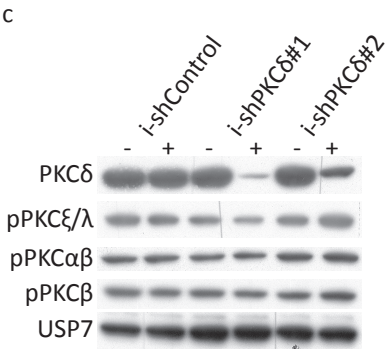

Supplement: Supplementary file 5 — Supplementary Figure 4 [file 41389_2018_41_MOESM5_ESM.pdf]
